# Alteration of osteoclast activity in childhood cancer survivors: Role of iron and of CB2/TRPV1 receptors

**DOI:** 10.1371/journal.pone.0271730

**Published:** 2022-07-21

**Authors:** Francesca Rossi, Chiara Tortora, Martina Di Martino, Alessandra Di Paola, Daniela Di Pinto, Maria Maddalena Marrapodi, Maura Argenziano, Elvira Pota

**Affiliations:** Department of Woman, Child and General and Specialist Surgery, University of Campania “Luigi Vanvitelli”, Napoli, Italy; Universite de Nantes, FRANCE

## Abstract

Childhood cancer survivors (CCS) are predisposed to the onset of osteoporosis (OP). It is known that iron overload induces osteoclasts (OCs) overactivity and that the iron chelator Deferasirox (DFX) can counteract it. The Cannabinoid Receptor type 2 (CB2) and the transient receptor potential vanilloid type-1 (TRPV1) are potential therapeutic targets for OP. In this study we isolated OCs from peripheral blood of 20 CCS and investigated osteoclast biomarkers expression and iron metabolism evaluating iron release by OCs and the expression of several molecules involved in its regulation. Moreover, we analyzed the effects of CB2 and TRPV1 stimulation in combination with DFX on osteoclast activity and iron metabolism. We observed, for the first time, an osteoclast hyperactivation in CCS suggesting a role for iron in its development. Moreover, we confirmed the well-known role of CB2 and TRPV1 receptors in bone metabolism, suggesting the receptors as possible key biomarkers of bone damage. Moreover, we demonstrated a promising synergism between pharmacological compounds, stimulating CB2 or inhibiting/desensitizing TRPV1 and DFX, in counteracting osteoclast overactivity in CCS to improve their quality of life.

## Introduction

The advancement of most pediatric malignancies curative therapy has determined an increase of childhood cancer survivors (CCS) population with high risk for several late effects that increase over time and interfere with the normal ageing process, resulting in a premature impairment of vital organs and tissues during adult-hood [1, 2]. Underling this condition, known as frailty, there is an alteration of the balance between pro-inflammation and anti-inflammation, which causes the onset of inflamm-aging, a low chronic proinflammatory status [3–5]. This condition can involve any organ and tissue. Therefore, tissues metabolically more active, such as bones, could be mostly vulnerable [6]. Young adult cancer survivors could develop low bone mineral density (BMD) consequent to bone metabolism alteration during childhood or adolescence, which may not allow the achievement of peak bone mass predisposing to osteoporosis (OP) of greater severity and earlier onset than in normal population [7–11]. Bone mineral alteration have been revealed after treatment for several pediatric malignancies [12–14]. The use of Prednisone and methotrexate, drugs commonly used for childhood leukemia treatment, significantly induce a decrease of BMD [15]. Bloomhardt *et al*. in a large cross-sectional study revealed a strong correlation between low lumbar spine BMD and post-therapy fractures in survivors of childhood leukemia/lymphoma 2 years off therapy [16]. It is well documented the relation between inflammation and iron. Interleukin-6 upregulate hepcidin expression that acts by binding ferroportin 1, an iron exporter, determining its internalization and degradation and consequently iron sequestration in cells [17–20]. Iron accumulation can significantly affect bone cells, promoting bone resorption [21]. In agreement, we previously revealed that iron overload, through the increase of the tartrate resistant acid phosphatase (TRAP), an important osteoclast biomarker, induces osteoclasts (OCs) overactivity and that iron chelators can decrease osteoclast activity [22, 23]. The Cannabinoid Receptor type 2 (CB2) and the transient receptor potential vanilloid type-1 (TRPV1), are potential therapeutic targets for bone diseases [24–26]. CB2 receptors has a key role in inhibiting OCs activity, while TRPV1 activation is stimulatory [27, 28]. CB2 stimulation inhibits the pro-osteoporotic effects determined by estrogens reduction in menopause [29] and by iron overload in thalassemia major [22]. Moreover, we also demonstrated an alteration of CB2 and TRPV1 receptors expression in OCs treated with Methylprednisolone and that drugs stimulating CB2 or inhibiting TRPV1 determine a decrease of OCs overactivation induced by MP suggesting their therapeutic use also for prevent glucocorticoid-induced bone mass loss [30]. In this study we investigated osteoclast activity in CCS and evaluated the possible influence of iron and CB2/TRPV1 receptors. We isolated OCs from peripheral blood mononuclear cells of 20 CCS and investigated osteoclast biomarkers expression and iron metabolism evaluating iron release by OCs and the expression of several molecules involved in its regulation. Then we analyzed the effects of CB2 and TRPV1 stimulation in combination or not with the iron chelator, DFX, on osteoclast activity and iron metabolism.

## Materials and methods

### Patients

Our study population included 20 healthy subjects (CTR) (12±4 years, 40% males) and 20 CCS (14±4 years, 50% males) among which 9 survivors of childhood Acute lymphoblastic leukemia (ALL), 4 survivors of childhood Hodgkin lymphoma (HL), 4 survivors of childhood Acute myeloid leukemia (AML) and 3 survivors of childhood Osteosarcoma. CCS and CTR were enrolled at the Department of Women, Child and General and Specialized Surgery of University of Campania Luigi Vanvitelli. The study was conducted according to the guidelines of the Declaration of Helsinki, the Italian National Legislation, and approved by the Ethics Committee of the University of Campania “Luigi Vanvitelli”, which formally approved the study (Identification code 266 18/09/2020). Written informed consents were obtained from participants’ parents and from patients themselves and assents were acquired before any procedures.

### Osteoclasts cell cultures

Primary cultures of OCs were obtained from the peripheral blood mononucleated cells (PBMCs) as previously described [31]. PBMCs were isolated by density gradient centrifugation (Histopaque 1077, Sigma Chemical, St Louis, MO) and diluted at 1×10^6^ cells/mL in α-Minimal Essential Medium (α-MEM) (Gibco, Uxbridge, UK) and supplemented with 10% fetal bovine serum (FBS) (Euroclone, Siziano, Italy), 100 IU/mL penicillin, 100 g/mL streptomycin (Gibco, Uxbridge, UK) and L-glutamine. Successively, PBMCs were plated in 24-well plates for 21 days in the presence of 25 ng/mL recombinant human macrophage colony-stimulating factor (rh-MCSF) (Peprotech, London, UK) and 50 ng/mL RANK-L (Peprotech, London, UK) to obtain differentiated OCs. Culture medium was replaced every three days. OCswere incubated at 37°C in a 5% CO_2_ humidified atmosphere and in normoxic conditions.

### Drugs and treatments

Osteoclasts were treated at day 21 (day in which they were full differentiated) with the CB2 agonist, JWH-133 [100 nM] and the TRPV1 agonist, Resinferatoxin, RTX [5 μM] (Tocris, Avonmouth, UK) for 48h or differentiated in the presence of DFX [5 μM] (Novartis S.p.a., Origgio, VA, Italy) from the first day of culture until day 21 and then treated for 48 h with JWH-133 and RTX. JWH-133 and RTX were dissolved in PBS containing DMSO, while DFX in sterile water. Final concentration of DMSO on cultures was 0.01%. Non-treated cultured cells were maintained in incubation media during the same treatment time with or without vehicle (DMSO 0.01%). The concentrations of JWH-133, RTX and DFX were decided after dose-response experiments, which revealed the concentrations responsible for strongest effects without altering cells viability (S1 Fig).

### Protein isolation, Western Blot

Proteins were extracted from OCs cultures using RIPA lysis and extraction buffer (Millipore, Italia). Protein concentration was determined through the Bradford dye-binding method (Bio-Rad, Hercules, CA, USA). The expression of TRAP, Cathepsin K, DMT1, TfR1, FPN-1, CB2 and TRPV1 proteins in total lysates from OCs cultures were revealed by Western Blot, by loading fifteen micrograms of proteins. Membranes were incubated overnight at 4°C with mouse monoclonal anti-TRAP (1:200 dilution; Santa Cruz SC-376875), rabbit monoclonal anti-Cathepsin K (1:1000 dilution; Abcam ab187647), mouse monoclonal anti-DMT1 (1:100 diluition; Santa Cruz sc-166884), rabbit monoclonal anti-TfR-1 (1:1000 dilution; abcam ab214039); rabbit polyclonal anti-FPN-1 (1:1000 dilution; Novus NBP1-21502), rabbit polyclonal anti-CB2 (1:500 dilution; Elabscience E-AB-65381), rabbit polyclonal anti-TRPV1 (1:1000 diluition; Novus NBP1-97417) and succesively with the specific secondary antibody for 1 h (Biorad Goat anti-rabbit IgG(H+L)-HRP conjugate 170–6515; Biorad Goat anti-mouse IgG(H+L)-HRP conjugate 170–6516). A C-DiGit blot scanner (LI-COR Biosciences) was used to detect reactive bands by chemiluminescence (Immobilion Western Millipore). To verify the protein loading it was used a mouse monoclonal anti-β-Actin antibody (1:100 dilution; Santa Cruz sc47778) as housekeeping protein. Images were captured and analyzed with “Image studio Digits ver. 5.0” software.

### Tartrate resistant acid phosphatase (TRAP) assay

TRAP was used as specific OCs activity marker and measured by using the ACP method (Takara Bio, Japan) as previously described [27]. Firstly, the citrate buffer pH 5.4, containing 60% acetone and 10% methanol, was used for 5 min at room temperature to fix cells. Successively, to each well of 24-well plate 50 μL of chromogen substrate solution (naphtol-AS-BI-phosphate substrate/fast red violet LB), mixed with 0.1 volume of sodium tartrate, was added. A red azoic dye with purplish red color product was obtained after TRAP enzyme cleavage os substrate and it was revealed with an optical microscope (Nikon Eclipse TS100, Nikon Instruments, Badhoevedorp, The Netherlands). TRAP positive and multinucleated-OCs count was made in at least three different wells in each group of patients and treatment. Therefore, in each experiment a positive and a negative control were included.

### Bone resorption assay

To perform the bone resorption assay, a commercially available kit was used (CosMo Bio, Tokyo, Japan) as previously described [31]. OCs were differentiated from PBMC in calcium phosphate coated 24 multiwell. RANK-L was added for OCs differentiation at 100 ng/ml concentration. DFX [5 μM] was added from the first day in culture, JWH-133 [100 nM] and RTX [5 μM] were added at day 12 for 48h. At day 14, 5% sodium hypochlorite was used to remove cells and to visualize and to count the reabsorption pits with an optical microscope (Nikon Eclipse TS100, Nikon Instruments, Badhoevedorp, The Netherlands).

### Iron assay

To determine iron (III) concentration, cell culture supernatants were collected after 48 hour-treatment. It was used the Iron Assay Kit (Abcam, Cambridge, UK) according to the manufacturer’s instructions. Firstly, standards and OCs supernatants were added into the well of 96-well plate and incubated with an acidic buffer to allow iron release. Successively, an iron probe at 25°C for 60 min was pipetted, protected from light. A colorimetric (593 nm) product was obtained after the reaction between released iron and chromogen and it was proportional to the iron amount. Tecan Infinite M200 (Tecan Group Ltd., Männedorf, Switzerland) spectrophotometer was used to measure the optical density at a wavelength of 593 nm. The determination of Iron (II) and Total Iron (II+III) contents of the test samples (nmol/μL) were obtained against a standard concentration curve. Iron (III) content can be determined as: Iron (III) = Total Iron (II+III) − Iron (II).

### ELISA assay

The evaluation of IL-6 concentration in cells cultures supernatants was determined by ELISA Assay using commercially available Human ELISA Kits (Invitrogen by Thermo Fisher, Waltham, MA, USA) according to the manufacturer’s instructions. Standards and supernatants were added into the wells of a microplate, coated with monoclonal antibodies specific to IL-6, and were run in duplicate. Enzyme-linked polyclonal antibodies specific for IL-6 was pipetted into the wells, after washing plate. The reaction was detected by the adjunct of a substrate solution. Tecan Infinite M200 (Tecan Group Ltd., Männedorf, Switzerland) spectrophotometer was used to determine the optical density at 450 nm. Cytokine concentration (pg/mL) was determined against a standard concentration curve.

### Statistical analysis

Results are showed as means ± standard deviation (SD). The experiments (TRAP assay and Bone Resorption assay) on cells were conducted in triplicate on each individual sample. Biochemical data were obtained from independent experiments (Western Blotting, Iron assay and ELISA) on each individual sample. To identify statistical differences among two group was performed a Student’s t test using Statgraphics Centurion 19 (Adalta; Statpoint Technologies). Instead, ANOVA test using Statgraphics Centurion 19 (Adalta; Statpoint Technologies) followed by a post hoc test was performed to identify statistical differences among more than two groups. Data are showed as mean ± SD. A p value ≤ 0.05 (*) was considered statistically significant.

## Results

### Characterization of OCs derived from CCS

**Fig 1:**
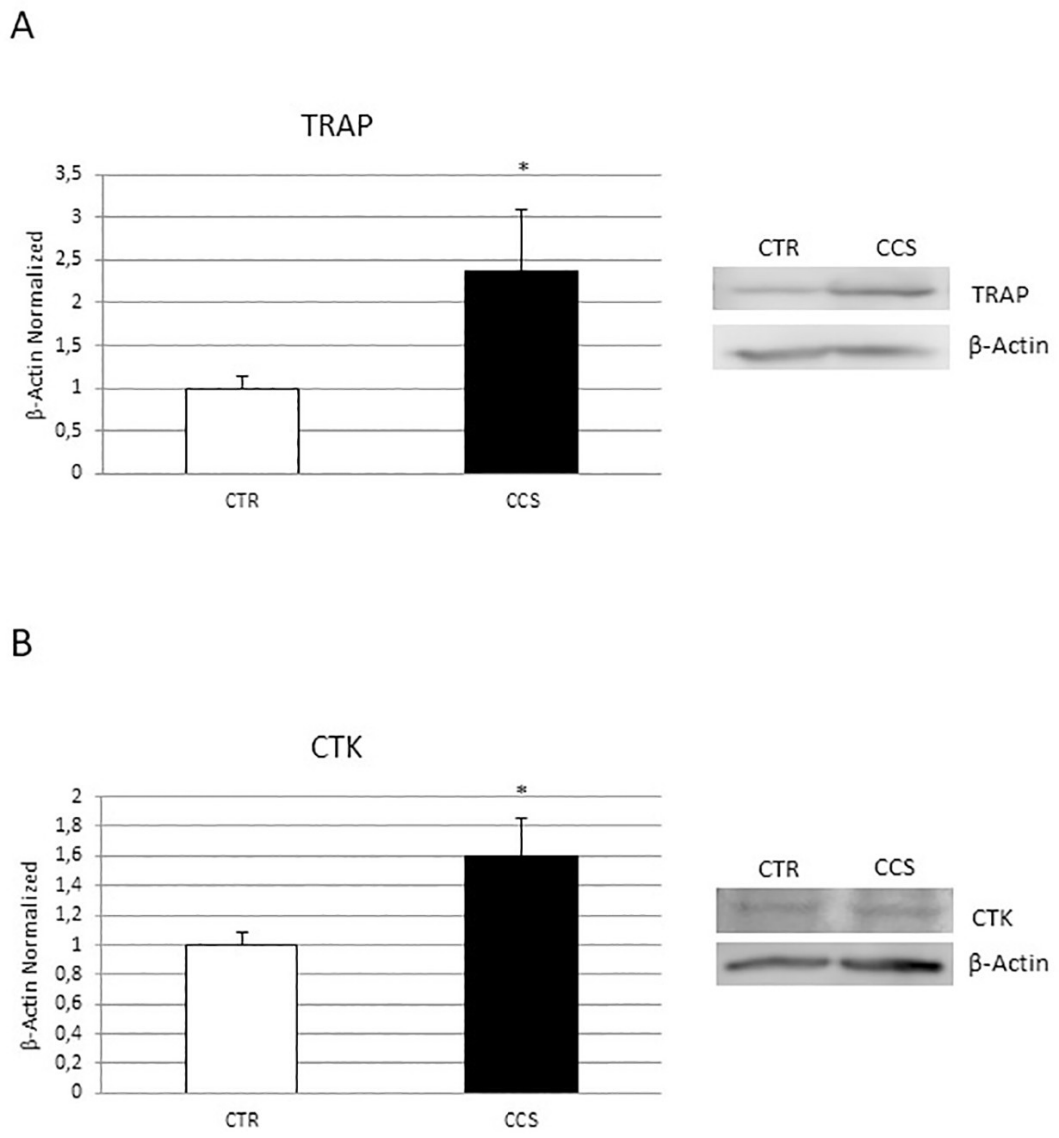
Characterization of OCs derived from CCS. TRAP (A), Cathepsin K (CTK) (B) protein expression in osteoclasts (OCs) from 5 Childhood cancer survivors (CCS) compared with OCs from 5 healthy subjects (CTR), determined by Western Blot starting from 15μg of total lysate. The most representative images are showed. The Image Studio Digits software has been used for the protein bands density detection. The intensity ratios of immunoblots compared to CTR, considered as 1, were quantified after normalization with the housekeeping protein β-Actin. Histograms represent the relative quantification for TRAP and CTK expression as mean ± SD of independent experiments on each individual sample. For statistical analysis it has been used a t-test. *Indicates p ≤ 0.05 compared to CTR.

**Fig 2:**
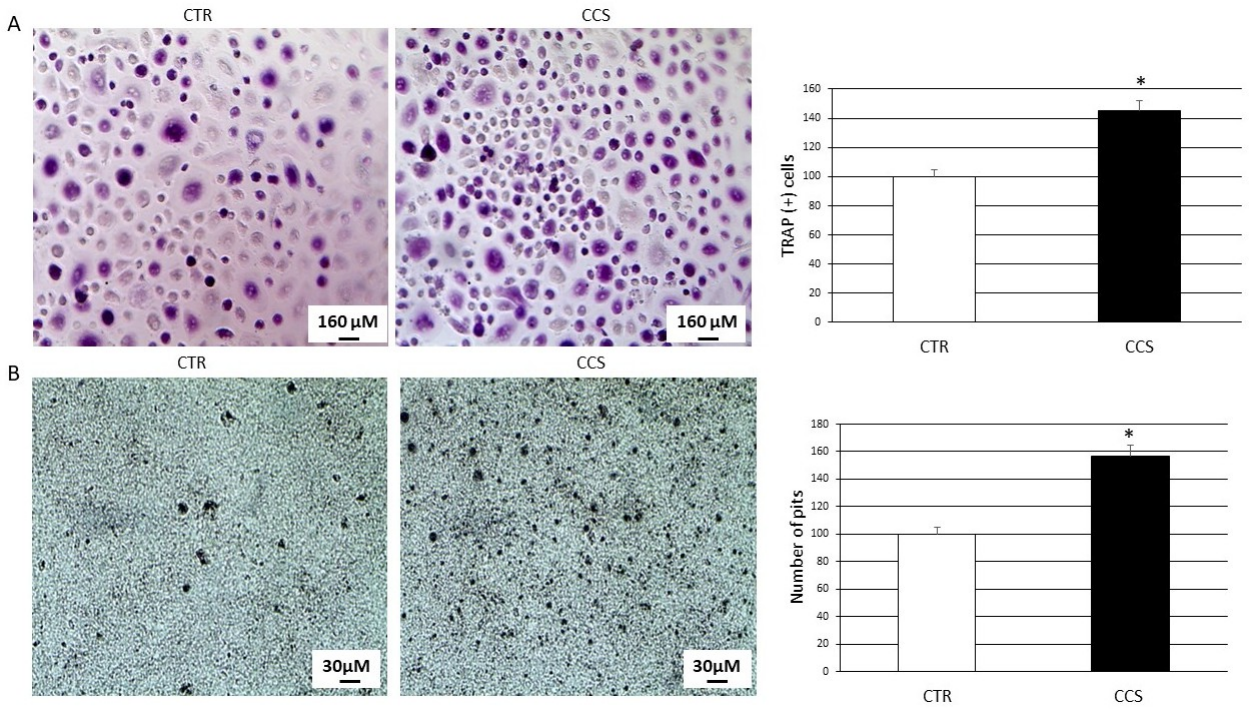
(A) TRAP assay on 5 CCS compared with OCs from 5 CTR. The most representative images are displayed (10x magnification). An AE2000 inverted microscope has been used to count TRAP (+) multinucleated (n ≥ 3) OCs (stained purple) in at least three different wells for each individual sample. Histogram shows the percentage number of TRAP (+) cells respect to the total cell number for each sample as mean ± SD. (B) Bone resorption assay on 5 CCS compared with OCs from 5 CTR. The most representative images are displayed (10x magnification). An AE2000 inverted microscope has been used to count the pits (represented in the image as darker areas) on the plate in at least three different wells for each individual sample. Histogram shows the percentage number of pits as mean ± SD. For statistical analysis it has been used a t-test. *Indicates p ≤ 0.05 compared to CTR.

We evaluated the expression of two important osteoclast markers TRAP and Cathepsin K (CTK) performing a Western Blot (WB) (Fig 1). Moreover, we evaluated osteoclast proliferation and activity performing TRAP assay and Bone Resorption assay (Fig 2). TRAP (Fig 1A) and Cathepsin K (CTK) (Fig 1B) were mostly expressed in OCs from CCS patients than in OCs from healthy subjects (CTR) suggesting an osteoclast hyperactivation. The TRAP assay (Fig 2A) revealed an increase of TRAP (+) cells in CCS and the bone resorption assay (Fig 2B), according to the biochemical data, an increased number of pits left by OCs after resorption.

### Evaluation of IL-6 and iron in OCs derived from CCS and effects of DFX on iron release

**Fig 3:**
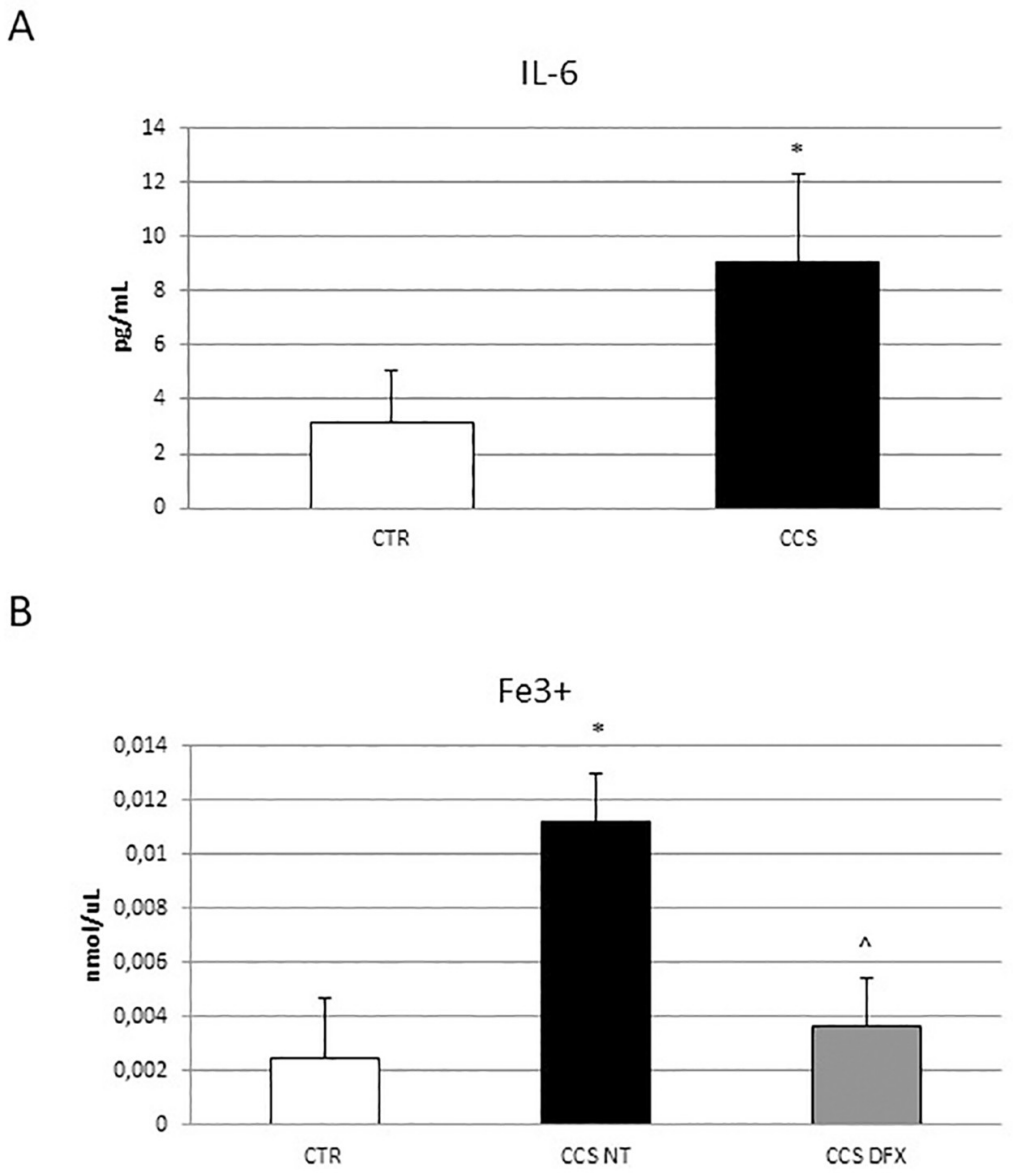
IL-6 and iron release in OCs derived from CCS. (A) Concentration of IL-6 (pg/mL) in 5 Childhood cancer survivors (CCS) compared to OCs from 5 healthy subjects (CTR) determined by ELISA Assay. Histogram shows concentration of IL-6 expressed as mean ± S.D. of independent experiments on each individual sample. Concentration of cytokine was calculated on a standard concentration curve. For statistical analysis it has been used a t-test. *Indicates p ≤ 0.05 compared to CTR. (B) Fe3+ intracellular concentrations (nmol/μL) in 5 Childhood cancer survivors (CCS) before and after Deferoxamine (DFX) [5μM] administration compared to OCs from 5 healthy subjects (CTR) detected by Iron Assay. Histogram shows concentration of Fe3+ expressed as the mean ± SD of independent experiments on each individual sample. For statistical analysis it has been used ANOVA test followed by a post hoc test. *Indicates p ≤ 0.05 compared to CTR. ^ indicates p ≤ 0.05 compared to CCS NT.

We evaluated the pro-inflammatory cytokine IL-6 release by enzyme-linked immunosorbent assay (ELISA) (Fig 3A) and measured the intracellular ferric iron ion concentration [Fe3+] by the colorimetric iron assay (Fig 3B). We demonstrated a statistically significant increase of IL-6 concentration in CCS-OCs with respect to CTR-OCs and an increase of [Fe3+] in OCs from CCS compared to CTR. These results let us to hypothesize a possible key role of inflammation and iron in CCS-OCs overactivation. Then we evaluated the effects of DFX on iron release by CCS-OCs (Fig 2B), demonstrating a significant reduction of iron concentration after DFX administration, thus confirming its iron chelating properties.

### Evaluation of iron metabolism in OCs derived from CCS

**Fig 4:**
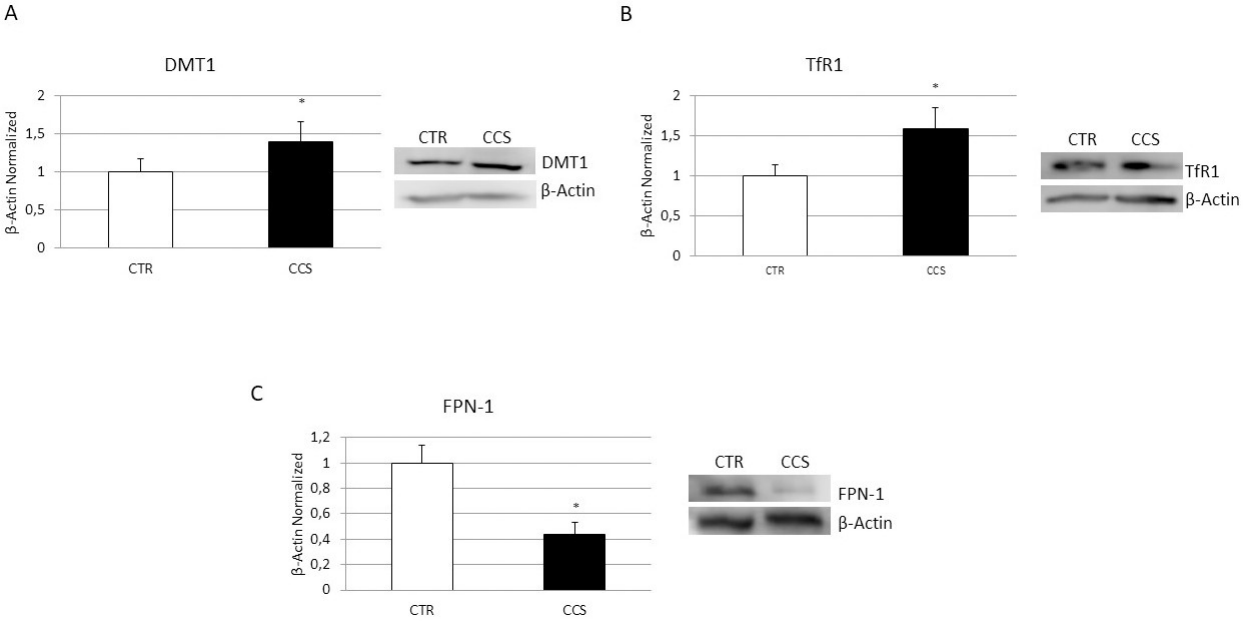
Iron metabolism in OCs derived from CCS. Protein expression of DMT1 (A), Transferrin Receptor 1, TfR1(B), Ferroportin, FPN1 (C) in OCs from 5 Childhood cancer survivors (CCS) compared with OCs from 5 healthy subjects (CTR), determined by Western Blot starting from 15μg of total lysate. The most representative images are showed. The Image Studio Digits software has been used for the protein bands density detection. The intensity ratios of immunoblots compared to CTR, considered as 1, were quantified after normalization with the housekeeping protein β-Actin. Histogram show the relative quantification for DMT1, TfR1 and FPN-1 as mean ± SD of independent experiments on each individual sample. For statistical analysis it has been used a t-test. *Indicates p ≤ 0.05 compared to CTR.

Successively, to strengthen our hypothesis about the role of iron in CCS-OCs overactivation, we performed several WB to analyze the expression levels of important modulators of iron metabolism (Fig 4), the iron transporter DMT1 (Fig 4A), the Transferrin receptor (TfR1) (Fig 4B), which mediates cellular iron uptake and the only known iron exporter protein, Ferroportin (FPN-1) (Fig 4C). We observed an increase of DMT1 and TfR1 and a reduction of FPN-1, confirming our hypothesis.

### CB2 and TRPV1 receptors expression in OCs derived from CCS

**Fig 5:**
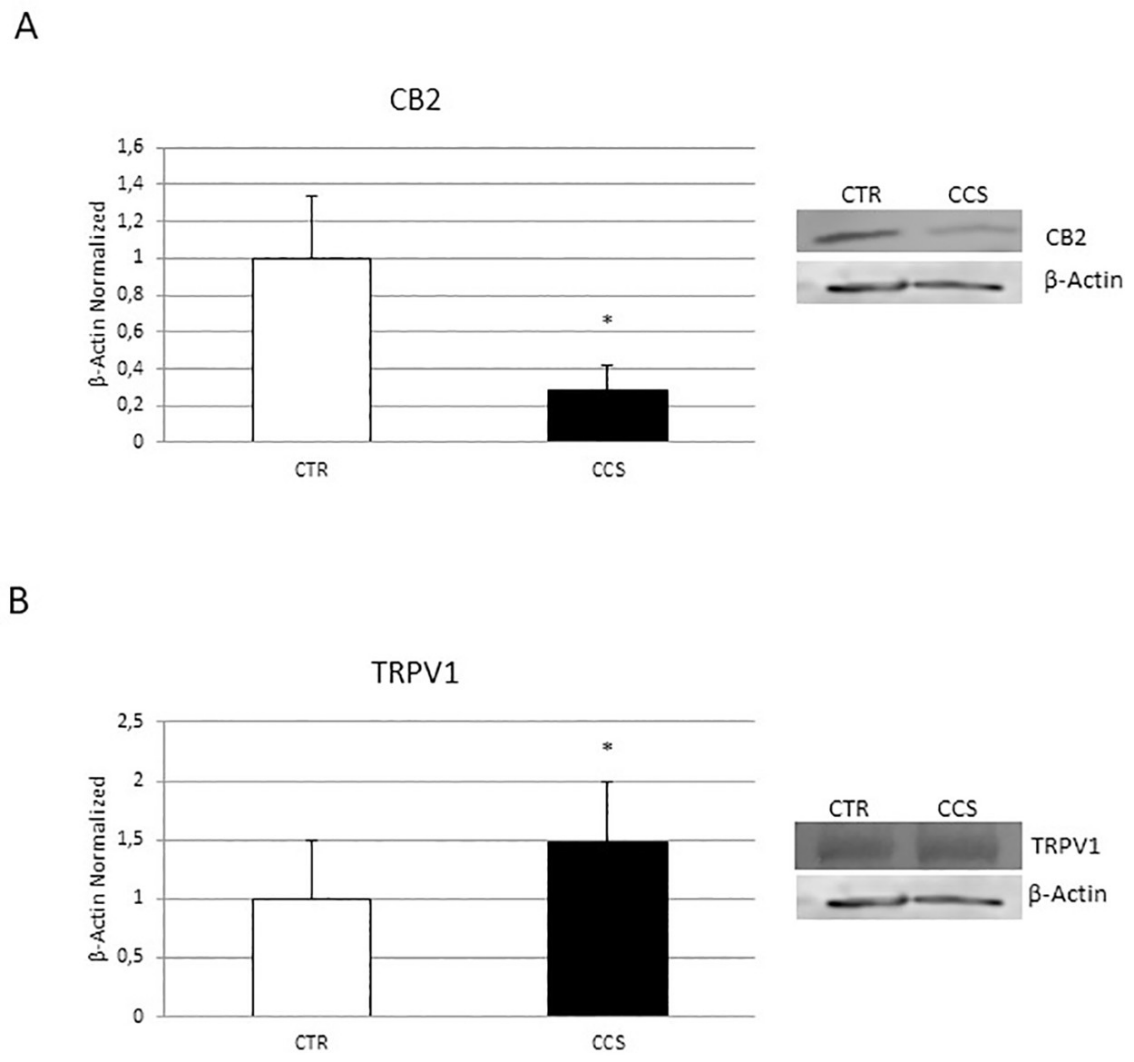
CB2 and TRPV1 expression in OCs derived from CCS. Protein expression of CB2 (A) and TRPV1 (B) in OCs from 5 Childhood cancer survivors (CCS) compared with OCs from 5 healthy subjects (CTR), determined by Western Blot starting from 15μg of total lysate. The most representative images are showed. The Image Studio Digits software has been used for the protein bands density detection. The intensity ratios of immunoblots compared to CTR, considered as 1, were quantified after normalization with the housekeeping protein β-Actin. Histogram shows the relative quantification for CB2 and TRPV1 expression as mean ± SD of independent experiments on each individual sample. For statistical analysis it has been used a t-test. *Indicates p ≤ 0.05 compared to CTR.

To understand the involvement of the CB2 and TRPV1 receptors in CCS-OCs overactivation, we performed a WB to analyze their expression levels (Fig 5). As expected, we observed a significant reduction of the CB2 receptor (Fig 5A), known to have an inhibitory effect on OCs activity, together with an increase of the pro-osteoporotic TRPV1 (Fig 5B). Considering these data, we then evaluated the effects of the stimulation of CB2 and TRPV1 with the respective specific agonists, JWH-133 and RTX for 48h, in CCS-OCs differentiated or not in presence of the iron chelator, DFX, on osteoclast activity and iron metabolism.

### Effects of treatments on osteoclast biomarkers expression

**Fig 6:**
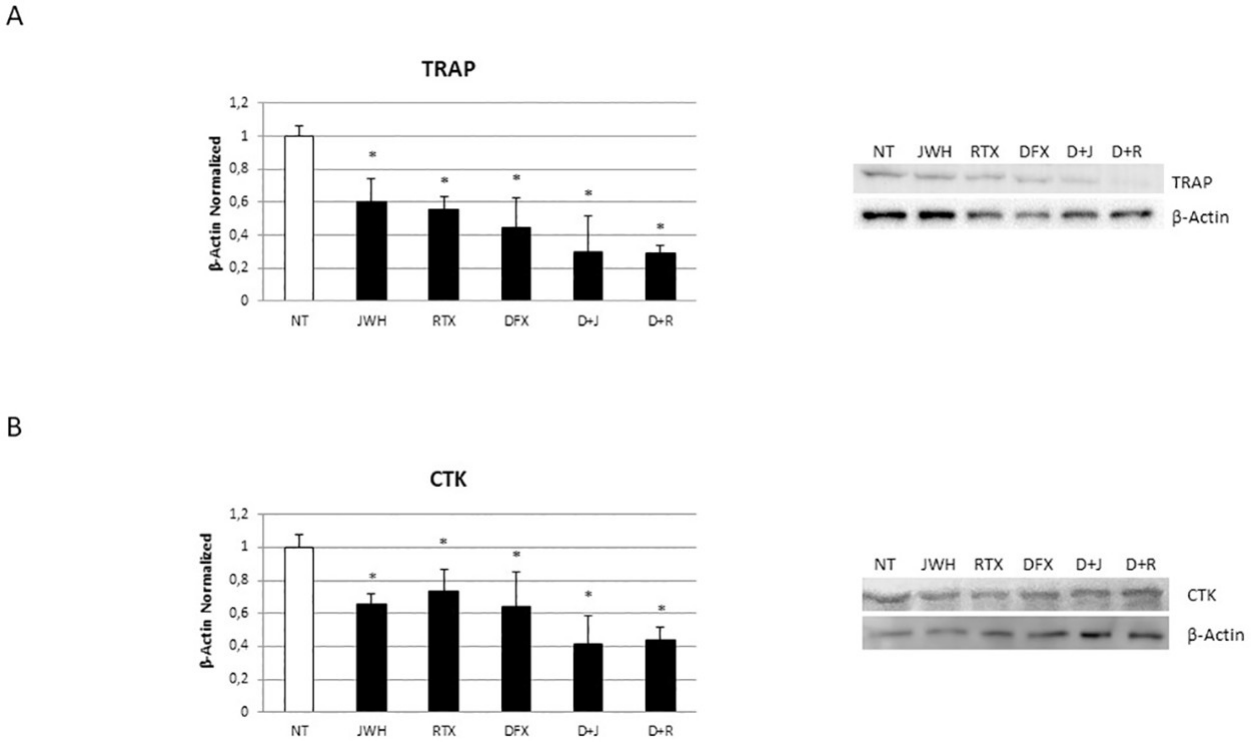
Effects of treatments on osteoclast biomarkers expression. Protein expression of TRAP (A) and Cathepsin K (CTK) (B) in OCs, differentiated or not in presence of DFX [5μM], from 5 Childhood cancer survivors (CCS) after 48h of exposure with JWH-133 [100nM] and RTX [5μM], determined by Western Blot starting from 15 μg of total lysate. The most representative images are showed. The Image Studio Digits software has been used for the protein bands density detection. The intensity ratios of immunoblots compared to CTR, considered as 1, were quantified after normalization with the housekeeping protein β-Actin. Histogram shows the relative quantification for TRAP and CTK as mean ± SD of independent experiments on each individual sample. For statistical analysis it has been used ANOVA test followed by a post hoc test. *Indicates p ≤ 0.05 compared to the untreated control (NT).

We treated *in vitro* CCS OCs with the CB2 agonist, JWH-133 [100 nM] and the TRPV1 agonist RTX [5μM] in combination or not with the iron chelator DFX [5 μM] and evaluated TRAP (Fig 6A) and CTK expression (Fig 6B) by WB. We observed a significant decrease of osteoclast biomarkers after both JWH-133 and RTX administration. The effect observed after TRPV1 stimulation with RTX suggest the activation and the consequent desensitization of the channel, as already demonstrate when there is a TRPV1 up-regulation. The chelator DFX alone induce a significant decrease of both TRAP and CTK. Interestingly, the pharmacological modulation of CB2 and TRPV1 in CCS-OCs differentiated in presence of DFX induces a greater reduction of TRAP and CTK.

### Effects of treatments on osteoclast number and activity

**Fig 7:**
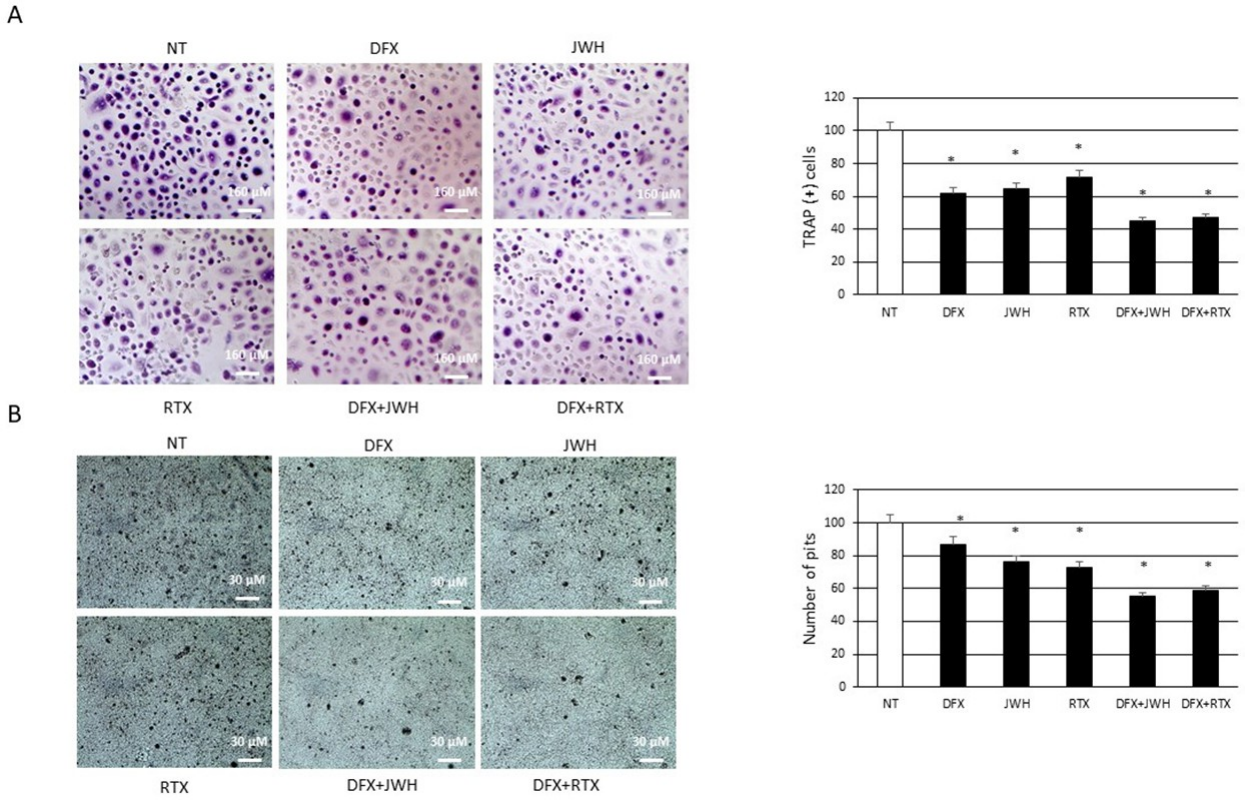
Effects of treatments on osteoclast number and activity. (A) TRAP assay on OCs, differentiated or not in presence of DFX [5μM], from 5 Childhood cancer survivors (CCS) after 48h of exposure with JWH-133 [100nM] and RTX [5μM]. The most representative images are showed (10x magnification). An AE2000 inverted microscope has been used to count TRAP (+) multinucleated (n ≥ 3) OCs (stained purple) in at least three different wells for each individual sample. Histogram shows the percentage number of TRAP (+) cells respect to the total cell number for each sample as mean ± SD. (B) Bone resorption assay on OCs, differentiated or not in presence of DFX [5μM], from 5 Childhood cancer survivors (CCS) after 48h of exposure with JWH-133 [100nM] and RTX [5μM]. The most representative images are displayed (10x magnification). An AE2000 inverted microscope has been used to count the pits (represented in the image as darker areas) on the plate in at least three different wells for each individual sample. Histogram shows the percentage number of pits as mean ± SD. For statistical analysis it has been used ANOVA test followed by a post hoc test. *Indicates p ≤ 0.05 compared to the untreated control (NT).

We evaluated the effects of JWH-133 [100 nM] and RTX [5 μM] on number and activity of CCS-OCs, differentiated or not in presence of DFX [5 μM], by TRAP assay (Fig 7A) and bone resorption assay (Fig 7B). TRAP assay and bone resorption assay revealed a significant reduction of CCS-OCs number and activity after both CB2 and TRPV1 modulation. In agreement with our previous findings, also DFX [5 μM] reduces the number of the TRAP (+) cells and the number of pits left by OCs after resorption. The reduction of CCS-OCs number and activity seems to be even more evident after the pharmacological modulation of CB2 and TRPV1 in combination with DFX.

### Effects of treatments on iron metabolism

**Fig 8:**
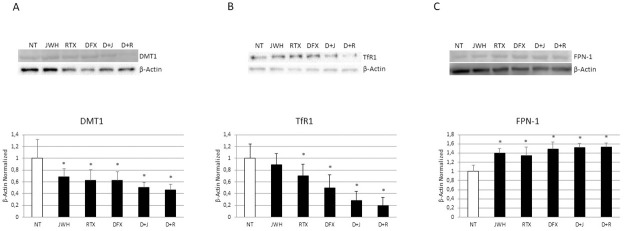
Effects of treatments on iron metabolism. Protein expression of DMT1 (A), Transferrin Receptor 1, TfF1(B), Ferroportin, FPN1 (C) in OCs, differentiated or not in presence of DFX [5μM], from 5 Childhood cancer survivors (CCS) after 48h of exposure with JWH-133 [100nM] and RTX [5μM], determined by Western Blot starting from 15 μg of total lysate. The most representative images are showed. The Image Studio Digits software has been used for the protein bands density detection. The intensity ratios of immunoblots compared to CTR, considered as 1, were quantified after normalization with the housekeeping protein β-Actin. Histogram shows the relative quantification for DMT1, TfR1, FPN1 expression is presented in histogram as mean ± SD of independent experiments on each individual sample. For statistical analysis it has been used ANOVA test followed by a post hoc test. *Indicates p ≤ 0.05 compared to the untreated control (NT).

We also evaluated by WB the effects of JWH-133 [100nM] and RTX [5 μM] on DMT1 (Fig 8A), TfR1 (Fig 8B) and FPN-1 (Fig 8C) expression in CCS-OCs differentiated or not in presence of DFX [5 μM]. We observed a reduction of DMT1 and TfR1 and a concomitant increase of FPN-1 after CB2 and TRPV1 modulation and after DFX administration. After the pharmacological modulation of CB2 and TRPV1 in CCS-OCs differentiated in presence of DFX [5 μM] we observed a more evident reduction of DMT1 and TfR1 and a greater increase of FPN-1.

## Discussion

Cancer children and adolescence patients could experience a strong alteration of bone metabolism, responsible for difficulties in achieving peak bone mass in proper manner and time. For this reason, young adult cancer survivors show high risk of low bone mineral density (BMD) and are predisposed to osteoporosis (OP) [7, 8, 16]. The cause of the BMD reduction has not been widely studied in childhood cancer survivors (CCS). Therefore, there is a growing need to understand the mechanisms involved in the development of OP in CCS to identify possible key biomarkers of bone damage and to identify treatments to correct this condition and its effects on CCS quality of life. In this study, we differentiated OCs from peripheral blood mononuclear cells of 20 CCS to investigate possible cause underlying the development of OP in CCS. For the first time in our knowledge, we observed in CCS an osteoclast hyperactivation as demonstrated by the overexpression of two important osteoclast markers, TRAP and Cathepsin K, and by the increased activity revealed through the bone resorption assay. We evaluated [Fe3+] concentration and the expression of key regulators of iron metabolism, as new possible causes of OCs hyperactivation. A dysregulation of iron homeostasis often occurs during inflammation [17] and in literature it is well documented the presence of a low-grade chronic inflammation in CCS [32, 33]. This status determines the involvement of local events and systemic activation of both innate and adaptive immune systems and induces tissue degeneration [34]. This persistent condition, characterized by increased release of pro-inflammatory cytokines, particularly IL-6, and inflammatory markers, could cause a decrease in circulating iron concentrations with an abnormal iron accumulation in the intracellular compartment [35, 36]. In agreement, Skou *et al*. observed elevated ferritin levels in childhood acute myeloid leukemia (AML) survivors [37]. High ferritin levels in diseases characterized by inflammation seem to limit inflammation [38]. During physiological processes, iron binds transferrin, its carrier protein, which recognizes the transferrin receptor (TfR) 1 on cells surface and determines iron entry into cells [39, 40]. Iron homeostasis is also guaranteed by hepcidin, that is up-regulated by the inflammatory cytokine, interleukin (IL)-6, through the IL-6R-JAK2-STAT3 pathway. Hepcidin binds the only known iron exporter protein FPN-1, determining its internalization and degradation and consequently iron sequestration in cells [20]. The observed TfR1 and DMT1 upregulation indicate iron high concentration in bone [41], that could significantly affect bone cells, promoting bone resorption [21]. Accordingly, we revealed an increase of IL-6, known to stimulate bone resorption [42], and of [Fe3+] in CCS-OCs compared to CTR-OCs together with an increase of DMT1 and TfR1 proteins, responsible for iron import, and a reduction of FPN-1, suggesting the involvement of iron in the CCS-OCs overactivation and confirming our previous findings. In 2014, we already demonstrated that iron overload causes an increase in TRAP expression levels, responsible for the consequent hyperactivation of OCs in β-thalassemia major. TRAP is directly related to ferritin levels and liver iron concentration (LIC), suggesting that iron chelation could have a role in OP therapy [22]. Deferoxamine (DFX) is used as an iron-chelator to reduce excess iron from the body [43]. By reducing excess iron, DFX counteracts the negative consequences induced by excess iron to multiple tissues, such as bone [44, 45]. It was reported that DFX counteract bone loss caused by several events, such as hindlimb unloading, ovariectomy, and ionizing radiation [45–48], therefore it could be a promising agent in counteract metabolic bone diseases. Cannabinoid Receptor type 2 (CB2) and Transient Receptor Potential Vanilloid type-1 (TRPV1) are proposed as possible therapeutic targets in bone diseases [24, 25]. We demonstrated that cannabinoid and vanilloid agonists modulate OCs activity in several forms of secondary OP [22, 29, 30]. Accordingly, we observed a significant up-regulation of the pro-osteoporotic TRPV1 and a concomitant reduction of the protective CB2 receptor in OCs from CCS compared to OCs from healthy donors. Considering this evidence, we evaluated the involvement of these receptors in the development of OP in CCS and their correlation with iron and chelating therapy. With this purpose, we treated CCS-OCs differentiated in presence of DFX, with CB2 and TRPV1 specific agonists, JWH-133 and RTX respectively, to evaluate their effects on OCs activity and iron metabolism. We confirmed DFX iron chelating properties also in CCS-OCs, observing a reduction in Fe3+ intracellular levels. We explained this result considering the decrease of DMT1 and TfR-1 and the concomitant increase of FPN-1, observed after DFX administration. Several studies already proved the role of DFX in reducing the iron uptake and increasing the iron export [49, 50]. As expected, the administered treatments induce a reduction of OCs activation affecting bone markers expression, active OCs number and bone resorption. As previously observed in other pathological conditions in which TRPV1 is upregulated, RTX induced its desensitization thus exerting the same effect of TRPV1 antagonism [51]. Interestingly, the effects of CB2 and TRPV1 modulation, after the iron chelation induced by DFX, are stronger, confirming the involvement of iron in OCs activation. Moreover, as already proved, this data suggests that CB2 and TRPV1 activation and desensitization could depend on iron [22]. The involvement of cannabinoid and vanilloid receptors in iron metabolism modulation has been recently investigated. CB2 activation seems to inhibit DMT1 expression and iron influx. Recently, also Jia *et al*. demonstrated that CB2 activation with JWH-133 inhibits DMT1 expression and iron influx [52]. Moreover, Seo *et*. *al*. demonstrated that the cannabinoid Δ9-THC blocks DMT1 phosphorylation thus inhibiting its function in HEK293 T cells [53]. It has been suggested that also calcium channels play an important role in iron uptake under condition of iron-overload. Treatments with calcium channels blocker decrease iron uptake and accumulation as demonstrated by in *in vivo* studies [54–57]. Accordingly with these results, we observed a reduction of DMT1 and TfR1 expression in CCS-OCs together with a significant increase of FPN-1, suggesting a role for CB2 and TRPV1 in modulating iron metabolism in iron overloaded CCS-OCs. This trend is more evident after the iron chelation induced by DFX, suggesting, for the first time in our knowledge, a synergism between the CB2 and TRPV1 agonists and the iron chelating agent DFX.

## Conclusions

Our study demonstrates, for the first time, an osteoclast hyperactivation in CCS and suggest a crucial role for iron in its development. Moreover, our study confirms the role of CB2 and TRPV1 receptors in bone metabolism modulation, suggesting the receptors as possible key biomarkers of bone damage. Moreover, our study demonstrates a promising synergism between pharmacological compounds, stimulating CB2 or inhibiting/desensitizing TRPV1 and DFX, in counteracting osteoclast overactivity in CCS to improve their quality of life. In the future *in vitro* and *in vivo* studies are needed to validate our data and to translate them to the clinical practice.

## Supporting information

S1 Fig Viability assay on CCS-OCs treated with different concentration of JWH-133, RTX and DFX. The viability of CCS-OCs was estimated by a cytofluorimetric assay after treatments. The histograms show results as cell number x 10^6^ and as mean ± SD of independent experiments on three different patients. For statistical analysis it has been used ANOVA test followed by a post hoc test. *Indicates p ≤ 0.05 compared to the untreated control (NT).
(JPG)

S2 Fig Uncropped figures. Proteins expression in Osteoclasts (OCs) from healthy subjects (CTR) and from Childhood cancer survivors (CCS) before and after treatments with JWH-133 [100nM] and RTX [5μM] in combination or not with DFX [5μM]. Proteins were determined by Western Blot starting from 15μg of total lysate. The protein bands were detected using Image Studio Digit Software and were quantified after normalizing with respective loading controls.
(PDF)
