# Supplementary figures and images for: Alteration of osteoclast activity in childhood cancer survivors: Role of iron and of CB2/TRPV1 receptors
Source: PLoS One. 2022 Jul 21;17(7):e0271730. doi: 10.1371/journal.pone.0271730 (PMC9302719; doi:10.1371/journal.pone.0271730)

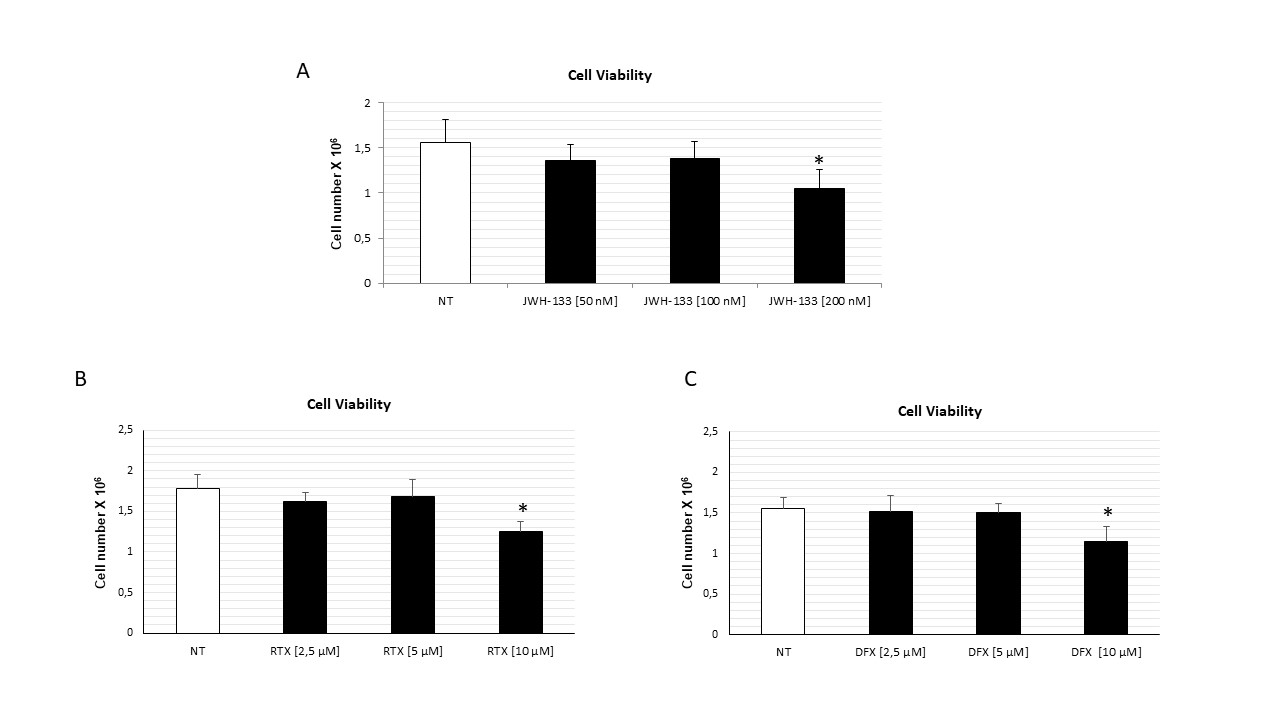

Supplement: S1 Fig — The viability of CCS-OCs was estimated by a cytofluorimetric assay after treatments. The histograms show results as cell number x 106 and as mean ± SD of independent experiments on three different patients. For statistical analysis it has been used ANOVA test followed by a post hoc test. *Indicates p ≤ 0.05 compared to the untreated control (NT). (JPG) [file pone.0271730.s001.jpg]
