# Supplementary material for: Alteration of osteoclast activity in childhood cancer survivors: Role of iron and of CB2/TRPV1 receptors
Source: PLoS One. 2022 Jul 21;17(7):e0271730. doi: 10.1371/journal.pone.0271730 (PMC9302719; doi:10.1371/journal.pone.0271730)
Supplement: S2 Fig — Proteins expression in Osteoclasts (OCs) from healthy subjects (CTR) and from Childhood cancer survivors (CCS) before and after treatments with JWH-133 [100nM] and RTX [5μM] in combination or not with DFX [5μM]. Proteins were determined by Western Blot starting from 15μg of total lysate. The protein bands were detected using Image Studio Digit Software and were quantified after normalizing with respective loading controls. (PDF) [file pone.0271730.s002.pdf]

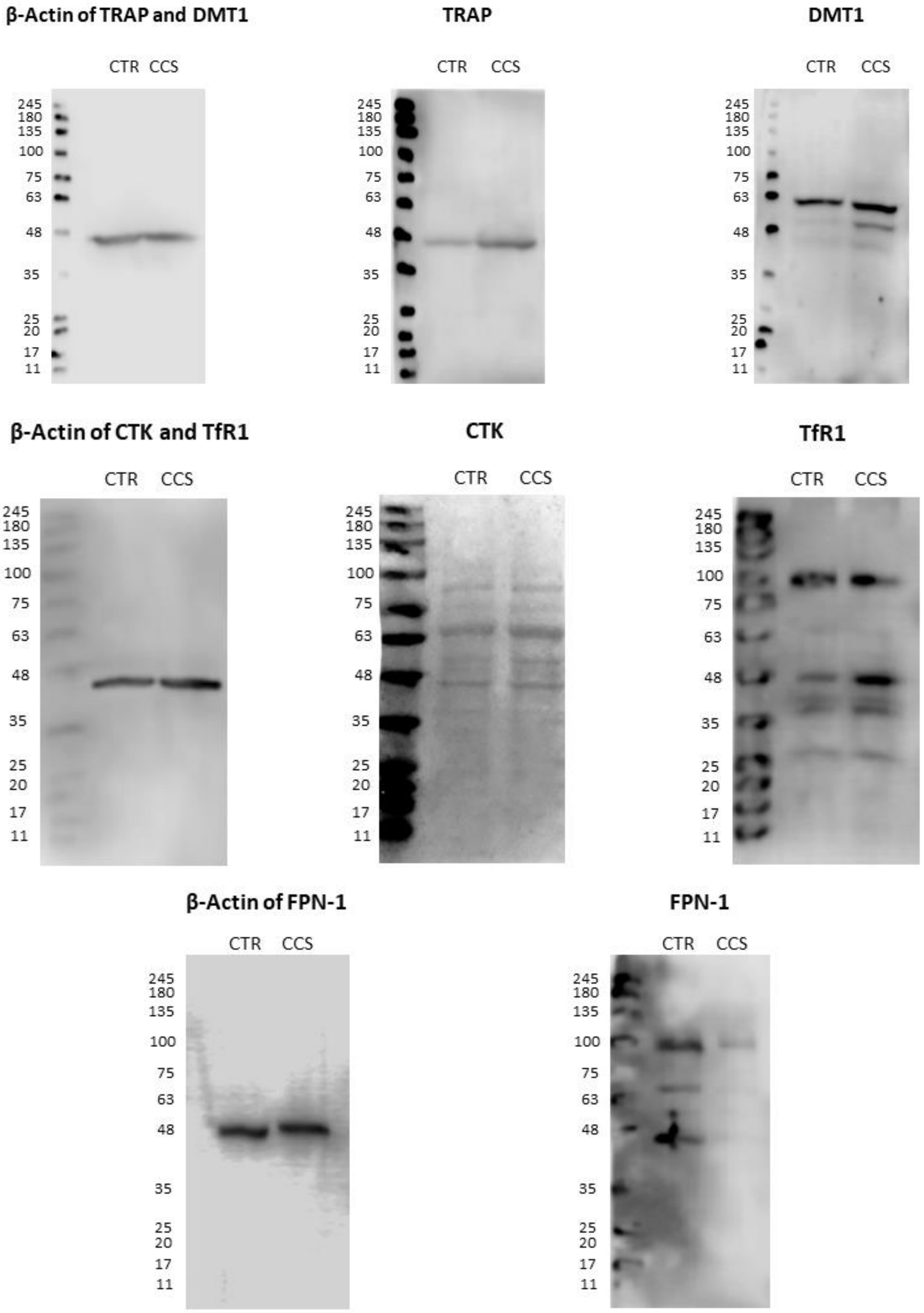
β-Actin of TRAP and DMT1
TRAP
DMT1
β-Actin of CTK and TfR1
CTK
TfR1
β-Actin of FPN-1
FPN-1
CTR
CCS
245
180
135
100
75
63
48
35
25
20
17
11

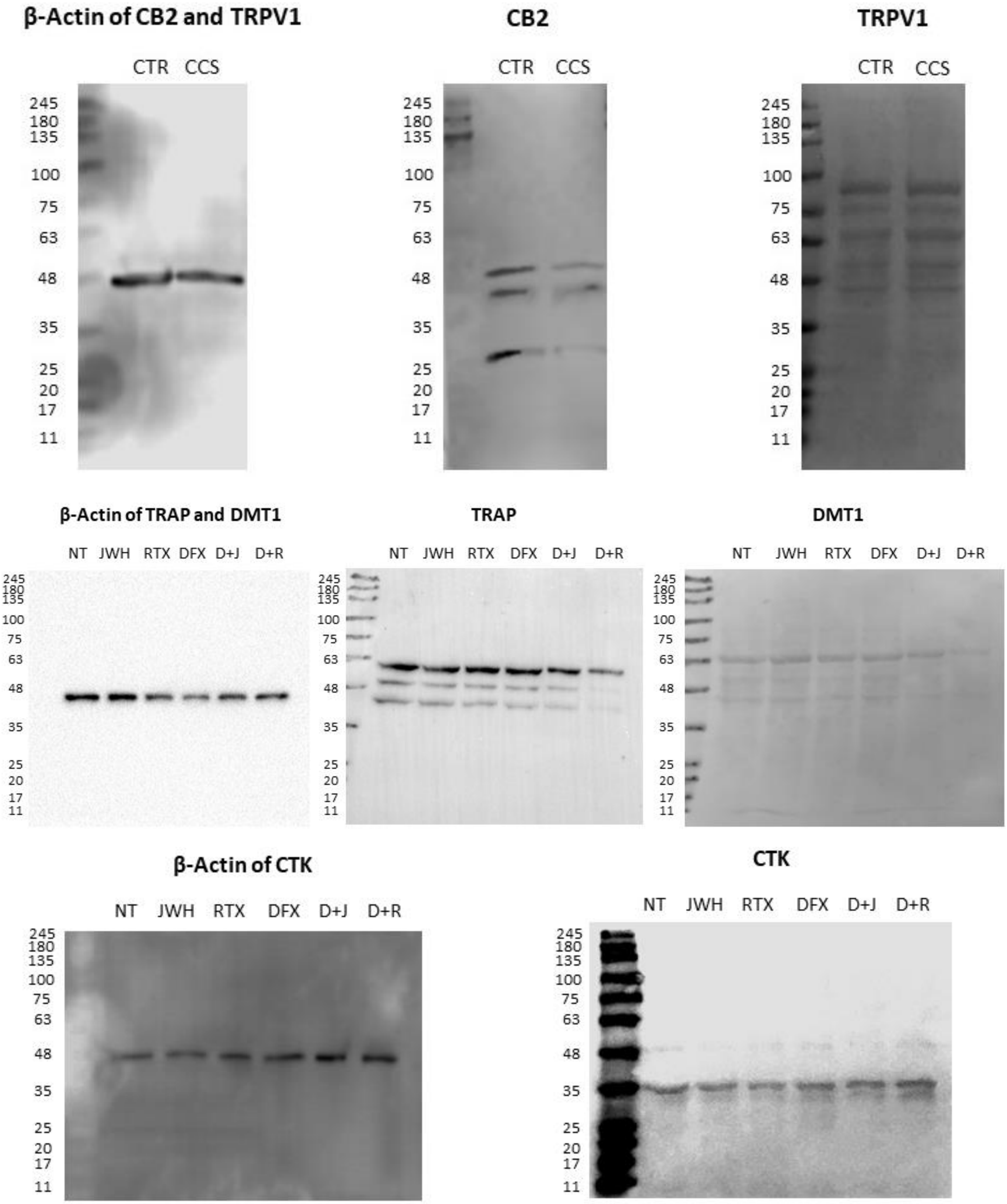
β-Actin of CB2 and TRPV1
CB2
TRPV1
CTR CCS
245 180 135 100 75 63 48 35 25 20 17 11
β-Actin of TRAP and DMT1
TRAP
DMT1
NT JWH RTX DFX D+J D+R
β-Actin of CTK
CTK

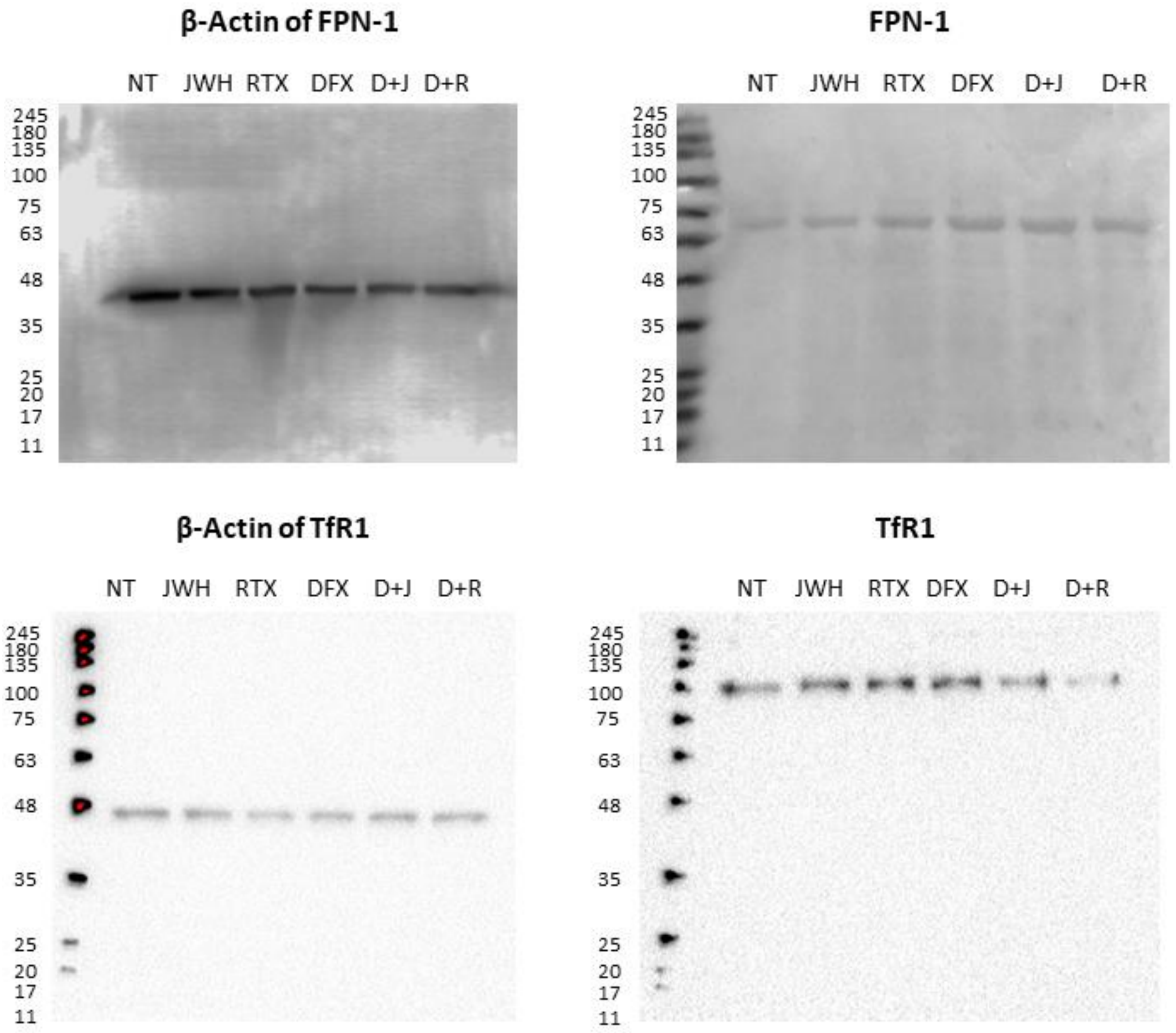
β-Actin of FPN-1
NT JWH RTX DFX D+J D+R
245 180 135 100 75 63 48 35 25 20 17 11
FPN-1
NT JWH RTX DFX D+J D+R
245 180 135 100 75 63 48 35 25 20 17 11
β-Actin of TfR1
NT JWH RTX DFX D+J D+R
245 180 135 100 75 63 48 35 25 20 17 11
TfR1
NT JWH RTX DFX D+J D+R
245 180 135 100 75 63 48 35 25 20 17 11
